# Supplementary material for: Cellular events of acute, resolving or progressive COVID-19 in SARS-CoV-2 infected non-human primates
Source: Nat Commun. 2020 Nov 27;11:6078. doi: 10.1038/s41467-020-19967-4 (PMC7695721; doi:10.1038/s41467-020-19967-4)
Supplement: Supplementary file 1 — Supplementary Information [file 41467_2020_19967_MOESM1_ESM.pdf]

Supplementary Table 1. Routes of exposure and viral inoculation

Animal ID	Exposure (dose)	Age	Sex	Species	Base Weight (kg)
GH99	Cumulative dose of 3.61×10^6 PFU including oral, intratracheal, intranasal, and conjunctival (both eyes)	13.9	Male	*Macaca mulatta*, Indian ancestry	15.5
HD09		12.87	Female	*Macaca mulatta*, Indian ancestry	7.7
NC40		16 (est)	Male	*Chlorocebus aethiops*	7.0
NC33		16 (est)	Female	*Chlorocebus aethiops*	3.7
HB37	Aerosol (approx. 2.0×10^3 $TCID_{50}$)	12.96	Male	*Macaca mulatta*, Indian ancestry	11.9
FR04		14.93	Male	*Macaca mulatta*, Indian ancestry	12.3
NC34		16 (est)	Female	*Chlorocebus aethiops*	4.3
NC38		16 (est)	Male	*Chlorocebus aethiops*	7.4

Est = estimated. Age of monkeys NC33, NC34, NC38, and NC40 are approximate.

Supplementary Table 2. Virus loads in clinical specimens

Week 1

Specimen	HB37	FR04	NC38	NC34	NC33	NC40	GH99	HD09
Bronchial Brush	4.99E+05	1.64E+04	5.81E+06	3.22E+07	4.65E+06	1.42E+06	1.26E+07	1.23E+04
Buccal Swab	3.67E+03	4.74E+04	ND	1.66E+06	ND	1.07E+04	I	2.55E+03
Nasal Swab	1.41E+06	1.52E+08	1.94E+04	6.45E+07	1.29E+05	1.05E+07	4.42E+04	1.42E+09
Pharyngeal Swab	5.03E+04	2.12E+06	2.47E+11	2.36E+09	7.00E+06	1.15E+08	I	6.54E+06
Rectal Swab	3.81E+03	9.90E+05	4.95E+03	4.32E+03	1.73E+07	6.22E+08	8.22E+03	ND
Vaginal Swab	NT	NT	NT	3.03E+05	5.16E+04	NT	NT	ND

Week 2

Specimen	HB37	FR04	NC38	NC34	NC33	NC40	GH99	HD09
Bronchial Brush	I	2.46E+03	2.16E+04	NT	2.92E+06	6.87E+03	1.95E+03	I
Buccal Swab	NT	1.71E+04	I	NT	6.40E+03	6.95E+03	3.78E+04	ND
Nasal Swab	NT	5.78E+06	2.07E+04	NT	3.27E+06	9.54E+04	1.03E+05	2.89E+03
Pharyngeal Swab	3.77E+04	3.11E+03	5.61E+04	NT	1.45E+03	9.21E+04	4.65E+03	I
Rectal Swab	2.63E+04	1.55E+08	4.69E+05	NT	3.93E+09	1.11E+05	2.71E+04	ND
Vaginal Swab	NT	NT	NT	NT	8.92E+04	NT	NT	ND

Week 3

Specimen	HB37	FR04	NC38	NC34	NC33	NC40	GH99	HD09
Bronchial Brush	ND	I	1.12E+05	NT	3.04E+07	1.41E+06	ND	ND
Buccal Swab	ND	I	I	NT	ND	ND	ND	ND
Nasal Swab	ND	7.53E+04	2.67E+04	NT	2.77E+05	1.42E+07	2.03E+04	4.13E+04
Pharyngeal Swab	NT	ND	ND	NT	1.05E+04	8.95E+04	I	4.28E+04
Rectal Swab	ND	1.31E+04	3.78E+06	NT	1.45E+04	3.45E+04	ND	ND
Vaginal Swab	NT	NT	NT	NT	I	NT	NT	ND

ND = Not Determined, NT = Not Tested due to lack of sample availability, I = Inconclusive.

Supplementary Table 3. Temperature and oxygen saturation.

	Baseline		*Week 1*		*Week 2*		*Week 3*	
**Animal ID**	**Temperature**	**SpO_2**	**Temperature**	**SpO_2**	**Temperature**	**SpO_2**	**Temperature**	**SpO_2**
FR04	100.4	98	100.9	93	101.1	95	102.3	94
GH99	100.4	99	101.5	97	100.3	92	100.8	93
HB37	100.9	91	102.6	94	102.2	95	103.5	96
HD09	101.1	98	100.7	95	101.3	93	101.3	94
NC33	100.3	-	99.5	96	100.1	96	99.4	92
NC34	99.9	96	100.9	90	-	-	-	-
NC38	99.9	94	102.2	96	101.2	95	100.8	91
NC40	102.1	94	101.4	97	101.4	98	102.1	94

Temperature measured in °F.

Supplementary Table 4. Comparison of disease outcomes by route of challenge or sex.

Aerosol vs. Multi-route (p-values adjusted)

Disease Factor	Baseline	Week 1	Week 2	Week 3	Necropsy
SpO_2	0.5743	0.2387	0.9998	0.9986	-
Temperature	0.7245	0.5392	0.7532	0.244	-
Viral Load	-	0.9025	0.9996	0.6114	-
Ranking	-	-	-		>.9999

Female vs. Male (p-values adjusted)

Disease Factor	Baseline	Week 1	Week 2	Week 3	Necropsy
SpO_2	0.8436	0.7922	0.9985	0.9969	-
Temperature	0.9809	0.1554	0.914	0.1556	-
Viral Load	-	0.514	0.987	0.0504	-
Ranking	-	-	-		0.1012

Data for rankings were based only on the necropsy time point. Two-tailed ANOVA were used for comparison of multiple groups followed by adjustment using Sidak's multiple comparisons test.

Supplementary Table 5. Correlations between viral loads from different specimens and disease ranking.

	Week 1			*Week 2*			*Week 3*		
**Specimen**	**R**	**P-value**	**P-value (adj)**	**R**	**P-value**	**P-value (adj)**	**R**	**P-value**	**P-value (adj)**
Bronchial brush	-0.71	0.058	0.754	-0.3	0.683	1	-0.5	1	1
Buccal swab	-0.1	0.95	1	0.2	0.917	1	-	-	-
Nasal swab	0.31	0.462	1	-0.14	0.803	1	0.43	0.419	1
Pharyngeal swab	-0.46	0.302	1	0.6	0.242	1	1	0.333	1
Rectal swab	0.36	0.444	1	-0.26	0.658	1	-	-	-
Vaginal swab	-	-	-	-	-	-	-	-	-

R = correlation coefficient. Missing statistical calculations are due to lack of sample availability or inconclusive results. Spearman's test was used for correlations followed by Dunn's Kruskal-Wallis' test for multiple comparisons.

Supplementary Table 6. Correlations between viral loads from bronchial brushes and the frequency and absolute number of monocyte subtypes.

Cell type	Week 1			Week 2			Week 3		
	R	P-value	P-value (adj)	R	P-value	P-value (adj)	R	P-value	P-value (adj)
% Classical monocytes	-0.9867	0.0133	0.0798	0.3823	0.5254	1	0.0409	0.9387	1
% Nonclassical monocytes	0.1767	0.8233	1	-0.2534	0.6809	1	-0.0162	0.9757	1
% Intermediate monocytes	0.9430	0.057	0.342	-0.5848	0.3004	1	-0.4343	0.3895	1
Absolute number classical monocytes	0.9393	0.0607	0.3642	-0.2921	0.6335	1	-0.1015	0.8483	1
Absolute number nonclassical monocytes	0.9627	0.0373	0.2238	-0.3379	0.5781	1	-0.0548	0.9179	1
Absolute number intermediate monocytes	0.9407	0.0593	0.3558	-0.3795	0.5287	1	-0.2807	0.5901	1

R = correlation coefficient. R and p-values were calculated using Pearson's correlation test, followed by adjusting for multiple comparisons using Bonferroni's test.

Supplementary Table 7. Levels of plasma chemokines.

Animal ID	Time Point	Eotaxin	IP-10	MCP-1	MCP-4	MDC	MIP-1a	MIP-1b	TARC
FR04	*Baseline*	32.01	547.00	67.13	13.04	238.50	18.65	46.56	0.95
	Week 1	26.75	542.34	62.31	12.45	289.45	23.62	51.40	0.94
	Week 2	33.64	455.34	60.43	7.55	278.59	19.06	41.03	0.65
	Week 3	18.17	196.14	31.44	2.79	189.15	4.23	23.66	0.39
	Necropsy	22.69	364.97	62.48	7.50	254.21	15.74	40.65	0.42
GH99	*Baseline*	44.71	482.21	60.30	28.10	184.46	22.66	153.92	1.05
	Week 1	54.40	1260.46	36.66	14.96	188.69	20.86	151.48	4.00
	Week 2	36.33	620.06	42.93	28.03	161.50	30.38	82.88	5.33
	Week 3	22.14	491.42	42.77	23.22	230.56	23.94	125.41	1.34
	Necropsy	33.72	452.24	57.13	20.75	326.46	19.89	143.02	1.33
HB37	*Baseline*	67.97	506.65	154.69	35.46	258.36	15.53	77.16	1.01
	Week 1	32.83	622.46	109.66	16.21	256.82	12.72	100.79	1.34
	Week 2	47.69	408.33	47.34	9.98	221.84	4.23	67.51	0.39
	Week 3	59.22	489.13	77.68	30.49	242.08	19.83	82.99	5.57
	Necropsy	45.32	630.00	104.97	16.66	518.60	13.95	105.09	1.34
HD09	*Baseline*	66.89	324.83	91.16	44.36	197.82	18.52	81.26	2.60
	Week 1	74.87	447.65	148.55	46.33	201.15	18.59	99.71	1.03
	Week 2	41.40	187.00	42.77	26.16	196.32	4.23	47.31	0.39
	Week 3	94.82	407.99	128.95	51.95	297.78	19.66	113.81	0.39
	Necropsy	66.19	541.69	93.97	34.73	371.65	17.53	104.06	1.39
NC33	*Baseline*	7.39	221.91	49.81	3.35	89.52	4.23	19.19	0.39
	Week 1	17.54	701.70	191.19	2.79	144.63	7.93	46.54	1.14
	Week 2	23.03	404.76	166.82	8.84	143.51	11.22	42.59	0.39
	Week 3	14.65	436.50	156.17	9.42	145.98	5.26	38.46	1.10
	Necropsy	13.78	185.31	295.92	2.79	71.28	4.23	56.30	0.39
NC34	*Baseline*	30.78	1303.10	123.93	3.45	119.71	16.19	45.48	2.44
	Week 1	88.83	7943.91	353.42	6.75	219.50	35.56	56.68	6.29
	Necropsy	116.82	78897.78	1606.75	6.00	300.12	29.53	232.02	6.23
NC38	*Baseline*	17.49	1357.14	123.61	8.60	122.65	15.16	48.62	2.42
	Week 1	12.33	766.14	161.39	3.35	239.25	24.47	46.26	2.46
	Week 2	13.22	1065.47	145.35	2.79	114.31	28.07	52.18	2.05
	Week 3	14.13	845.43	132.72	5.21	107.54	14.18	52.82	0.69
	Necropsy	31.00	794.69	184.65	15.35	156.91	28.71	33.34	0.79
NC40	*Baseline*	35.58	875.23	87.90	9.15	114.59	32.46	26.43	1.50
	Week 1	39.70	1498.56	194.17	9.45	103.31	41.35	52.20	2.43
	Week 2	41.68	1703.38	270.96	15.97	134.91	122.34	47.64	2.30
	Week 3	41.24	1014.66	239.48	16.33	113.86	64.80	45.91	2.03
	Necropsy	37.49	1611.59	185.71	15.12	149.16	60.97	49.18	3.08

Values are shown in pg/mL

Supplementary Table 8. Log_2 fold change of chemokines relative to baseline.

Animal ID	Time Point	Eotaxin	IP-10	MCP-1	MCP-4	MDC	MIP-1a	MIP-1b	TARC
FR04	*Week 1*	-0.26	-0.01	-0.11	-0.07	0.28	0.34	0.14	-0.02
	Week 2	0.07	-0.26	-0.15	-0.79	0.22	0.03	-0.18	-0.55
	Week 3	-0.82	-1.48	-1.09	-2.23	-0.33	-2.14	-0.98	-1.29
	Necropsy	-0.50	-0.58	-0.10	-0.80	0.09	-0.24	-0.20	-1.17
GH99	*Week 1*	0.28	1.39	-0.72	-0.91	0.03	-0.12	-0.02	1.92
	Week 2	-0.30	0.36	-0.49	0.00	-0.19	0.42	-0.89	2.34
	Week 3	-1.01	0.03	-0.50	-0.28	0.32	0.08	-0.30	0.35
	Necropsy	-0.41	-0.09	-0.08	-0.44	0.82	-0.19	-0.11	0.34
HB37	*Week 1*	-1.05	0.30	-0.50	-1.13	-0.01	-0.29	0.39	0.40
	Week 2	-0.51	-0.31	-1.71	-1.83	-0.22	-1.88	-0.19	-1.38
	Week 3	-0.20	-0.05	-0.99	-0.22	-0.09	0.35	0.11	2.46
	Necropsy	-0.58	0.31	-0.56	-1.09	1.01	-0.15	0.45	0.40
HD09	*Week 1*	0.16	0.46	0.70	0.06	0.02	0.01	0.30	-1.34
	Week 2	-0.69	-0.80	-1.09	-0.76	-0.01	-2.13	-0.78	-2.74
	Week 3	0.50	0.33	0.50	0.23	0.59	0.09	0.49	-2.74
	Necropsy	-0.02	0.74	0.04	-0.35	0.91	-0.08	0.36	-0.90
NC33	*Week 1*	1.25	1.66	1.94	-0.27	0.69	0.91	1.28	1.55
	Week 2	1.64	0.87	1.74	1.40	0.68	1.41	1.15	0.00
	Week 3	0.99	0.98	1.65	1.49	0.71	0.31	1.00	1.49
	Necropsy	0.90	-0.26	2.57	-0.27	-0.33	0.00	1.55	0.00
NC34	*Week 1*	1.53	2.61	1.51	0.97	0.87	1.13	0.32	1.37
	Necropsy	1.92	5.92	3.70	0.80	1.33	0.87	2.35	1.35
NC38	*Week 1*	-0.50	-0.82	0.38	-1.36	0.96	0.69	-0.07	0.02
	Week 2	-0.40	-0.35	0.23	-1.63	-0.10	0.89	0.10	-0.24
	Week 3	-0.31	-0.68	0.10	-0.72	-0.19	-0.10	0.12	-1.81
	Necropsy	0.83	-0.77	0.58	0.84	0.36	0.92	-0.54	-1.62
NC40	*Week 1*	0.16	0.78	1.14	0.05	-0.15	0.35	0.98	0.69
	Week 2	0.23	0.96	1.62	0.80	0.24	1.91	0.85	0.62
	Week 3	0.21	0.21	1.45	0.84	-0.01	1.00	0.80	0.44
	Necropsy	0.08	0.88	1.08	0.72	0.38	0.91	0.90	1.04

Supplementary Table 9. Rotation table derived from PCA plots.

	PC1	PC2	PC3	PC4	PC5	PC6	PC7	PC8
Eotaxin	0.51349854	0.14431892	0.04547296	0.10908504	-0.2471479	0.22538446	-0.6843669	-0.3482608
IP-10	-0.2522775	0.55999126	-0.0765231	0.02243312	0.41068394	-0.1618914	0.05487606	-0.6469353
MCP-1	0.11727127	0.51945467	0.4952681	0.25731776	0.20002956	0.44221279	0.14981145	0.38328048
MCP-4	0.51986265	-0.0023534	0.07196805	-0.2206943	-0.2665758	0.11724746	0.67825244	-0.3619612
MDC	0.45844363	-0.0508976	0.21350047	0.3735466	0.24485256	-0.7283914	0.0253733	0.10473167
MIP-1a	0.15406887	0.2517465	-0.7797945	0.47174206	-0.1062296	0.09007799	0.16325863	0.19030037
MIP-1b	0.39295357	-0.0473738	-0.2926367	-0.510066	0.65448152	0.14743768	-0.1017211	0.19263585
TARC	0.01858282	0.57230412	-0.0480451	-0.5004337	-0.3994979	-0.39152	-0.0922837	0.31301387

All elements used for the Principal Component (PC) Analysis are shown. PC = PCA Coefficients for each case (row = each chemokine) on each factor (column) that can be plotted. The square of each PC defines the amount of variation contributed by each variable within the factor.

Supplementary Table 10. Antibodies used for flow cytometry and immunohistochemistry.

Flow cytometry antibodies

Marker	Fluorochrome	Clone	Ref.	Company	Known Reactivity
PD-1	APC	EH12.2H7	329908	Biolegend	RM & AGM
CD56	Alexa 700	B159	557919	BD	RM _ AGM not reported
CD183 (CXCR3)	APC/Cyanine7	G025H7	353722	Biolegend	RM & AGM
CD196 (CCR6)	BV605	G034E3	353420	Biolegend	RM & AGM
CD4	BV797	L200	563914	BD	RM & AGM
CD3	PE	SP34-2	552127	BD	RM & AGM
CD3	BV650	SP34-2	563918	BD	RM & AGM
CD20	BV650	2H7	563780	BD	RM & AGM
CD95	PE/Cyanine5	DX2	559773	BD	RM & AGM
CD8a	Texas Red	RPA-T8	MHCD0817	Life technology	RM & AGM
CD159a (NKG2a)	PE/Cyanine7	Z199	B10246	Beck Coulter	RM & AGM
CD16	BV711	3G8	563127	BD	RM & AGM
CD16	BUV496	3G8	564653	BD	RM & AGM
CD86	FITC	FUN-1	557343	BD	RM _ AGM not reported
CD11c	APC	3.9	17-0116-42	eBioscence	RM & AGM
HLA-DR	APC/Cyanine7	L243	307618	Biolegend	RM & AGM
CD14	V450	M5E2	561390	BD	RM & AGM
CD163	BV711	GHI/61	563889	BD	RM _ AGM not reported
CD184 (CXCR4)	PECF594	12G5	562389	BD	RM & AGM
CD45	BV786	D058-1283	563861	BD	RM & AGM
CX3CR1	PE	K0124E1	355704	Biolegend	RM _ AGM not reported
CD11b	PE/Cyanine5	ICRF44	301308	Biolegend	RM & AGM
CD206	PE/Cyanine7	19.2	25-2069-42	BD	RM & AGM

Immunohistochemistry antibodies

Primary Antibody	Species	Company	Ref.	Dilution	Secondary Antibody
CD206	Goat	R&D	AF2535	1:50	Donkey anti-goat 647 (far red)
CD11b	Rabbit	Abcam	Ab133357	1:20	Donkey anti-rabbit 488 (green)
CD16	Mouse IgG2a	Novocastra	NCL-CD16	1:40	Permanent Red
DAPI	Dye - N/A	Invitrogen	D1306	1:20,000	N/A
MPO	Rabbit	Dako	A0398	1:6000	Permanent Red
CD3	Rabbit	Dako	A0452	1:50	Goat anti-rabbit 488 (green)
CD68	Mouse IgG1	Dako	M0814	1:20	Goat anti-IgG1 568 (red)
CD163	Mouse IgG1	Leica	NCL-L-CD163	1:50	Goat anti-IgG1 568 (red)

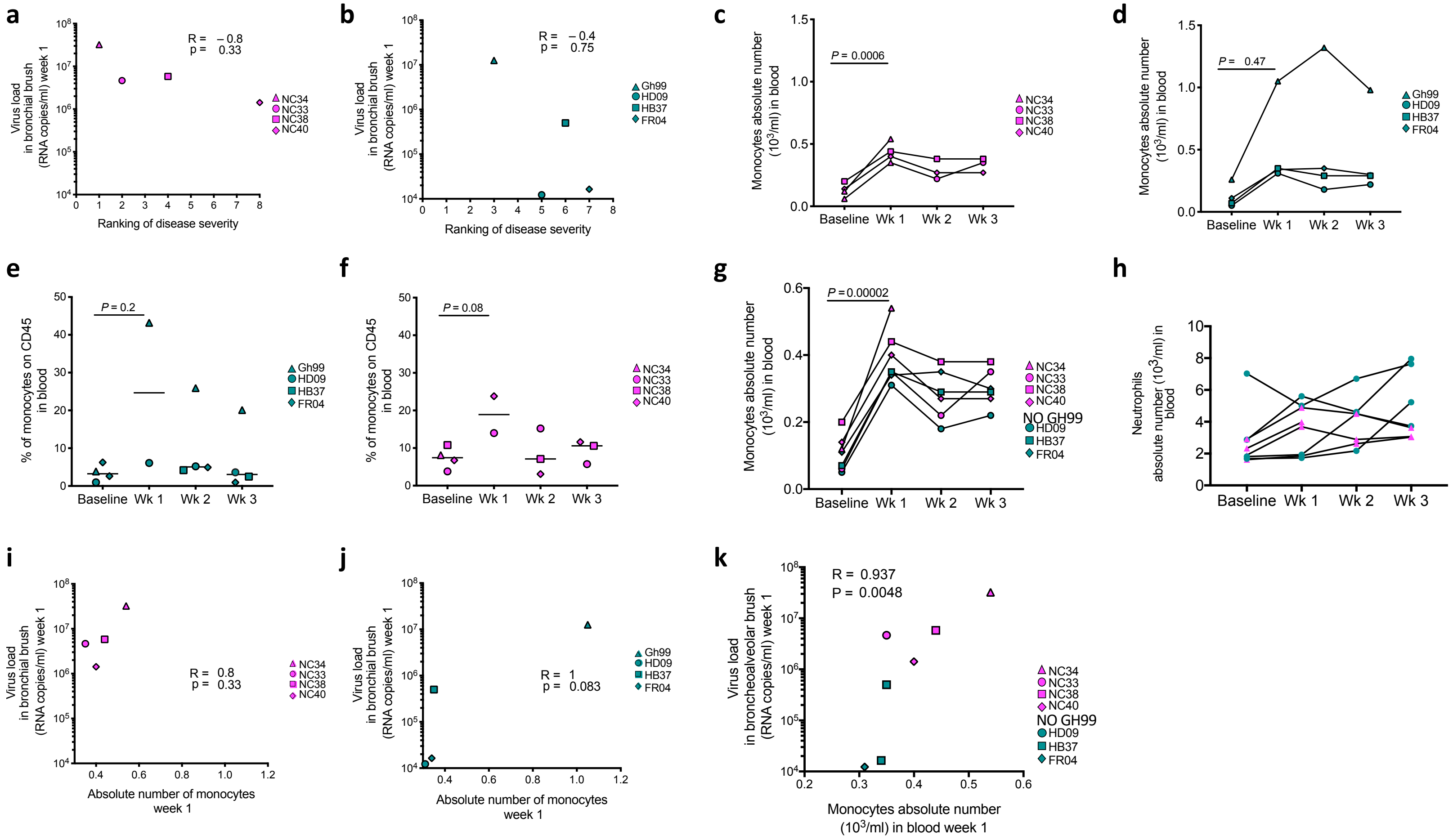

Supplementary Figure 1. Kinetics of monocytes in rhesus macaques and African green monkeys. Association between SARS-CoV-2 load in bronchial brushes and ranking of disease severity in AGM (**a**) and RM (**b**) (most severe case = 1, mildest case = 8). Changes in monocyte count in blood before and after infection in AGM (**c**) and RM (**d**). Proportion of total monocytes (HLA-DR+ CD16+ and/or CD14+ out of CD45+ leukocytes) in PBMCs from AGM (**e**) and RM (**f**) over time. (**g**) Absolute number of monocytes and neutrophils (**h**) in blood over time. Correlation between SARS-CoV-2 viral load in bronchial brushes and absolute count of monocytes in blood at one-week post-infection (1wk pi) in AGM (**I**) and RM (**j**). (**k**) Correlation between SARS-CoV-2 viral load in bronchial brushes and absolute count of monocytes excluding GH99 (1wk pi). Spearman test was used for correlations and Kruskal -Wallis test, Dunn's multiple comparison in **c - g**.

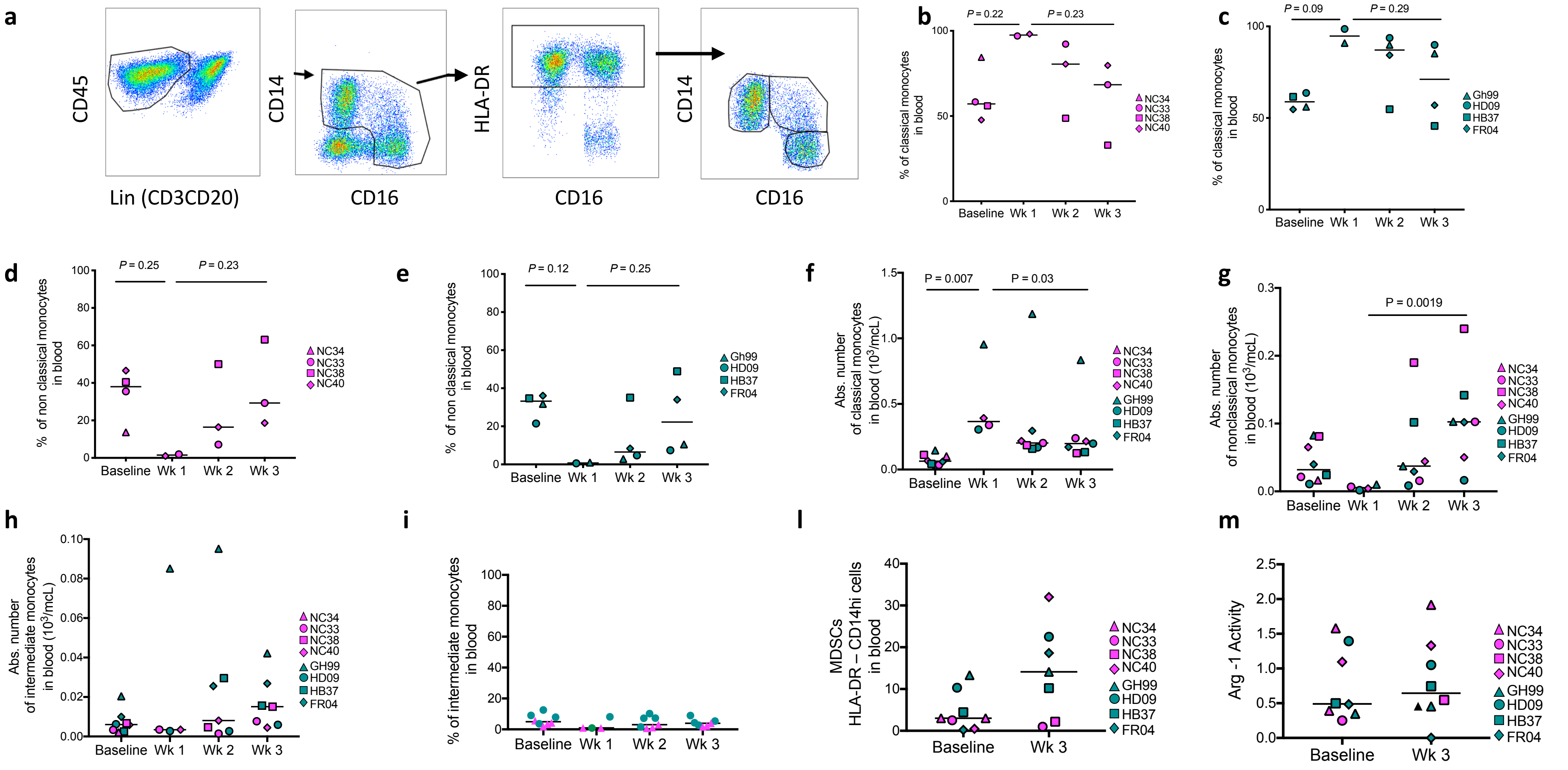

Supplementary Figure 2. SARS-CoV-2 associated changes in monocytes subsets in blood. (**a**) Gating strategy highlighting classification of classical (CD14hi CD16low), non-classical (CD14low CD16hi), and intermediate (CD14hi CD16hi) monocytes from blood. Percent of (**b**) classical monocytes out of total monocytes in PBMCs from AGM or RM (**c**). Percent of non-classical monocytes out of total monocytes in PBMCs in AGM (**d**) or RM (**e**). Absolute number of classical (**f**), nonclassical (**g**), and intermediate (**h**) monocytes in blood over time. (**i**) Percent of intermediate monocytes out of total monocytes in PBMCs over time. (**l**) Percent of MDSCs (HLA-DR- CD14hi) cells in blood out of live/CD45+ cells. (**m**) Arg-1 activity (unit/ml) measured at baseline or three weeks post-infection. Spearman test was used for correlations and Kruskal -Wallis test, Dunn's multiple comparison in **c - g**.

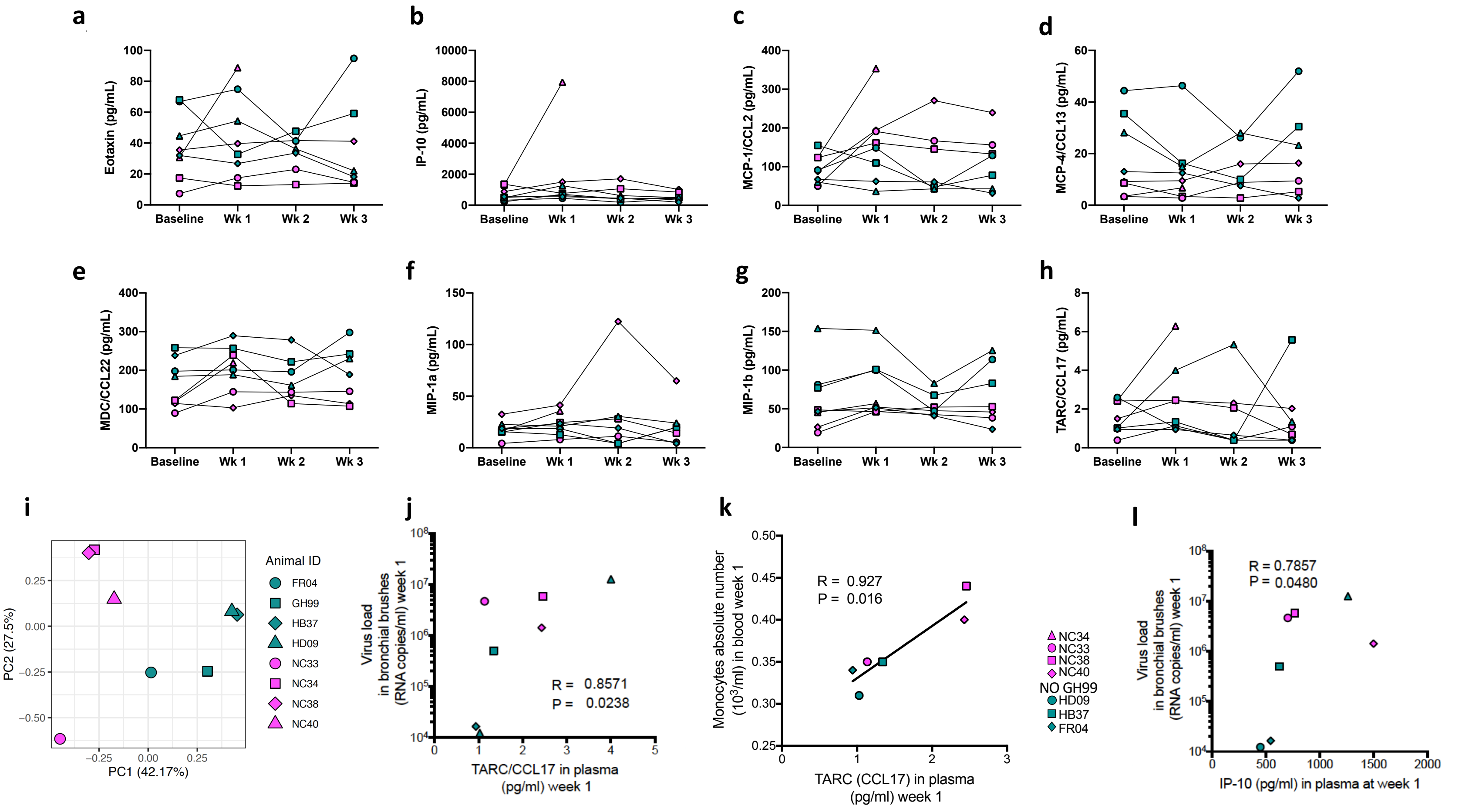

Supplementary Figure 3. **Chemokines promote cell migration of monocytes from the blood to the lung following infection**. Levels of chemokines in the peripheral blood over time, including (**a**) Eotaxin, (**b**) IP-10, (**c**) MCP-1 (CCL2), (**d**) MCP-4 (CCL13), (**e**) MDC (CCL22), (**f**) MIP-1a, (**g**), MIP-1b, and (**h**) TARC (CCL17). (**i**) Principal component analysis (PCA) plot where each point represents an individual animal and points are colored by species (AGM = magenta and RM = teal). Baseline raw data for the eight chemokines shown in a-h were used to calculate the variance between individuals that determined the position of each individual point on the plot. Principal components 1 and 2 (PC1 and PC2) explain approximately 70% of the total variance between individuals and individuals which are more closely related are clustered nearer to each other. (**j**) Correlation between TARC (CCL17) levels in blood and SARS-CoV-2 viral load in bronchial brushes one week post-infection. (**k**) Correlation between TARC (CCL17) levels in blood and the absolute number of monocytes at one week post infection. (**l**) Correlation between IP-10 levels in blood and SARS-CoV-2 viral load in bronchial brushes one week post-infection. The Spearman test was used in **j-l** (n = 7).

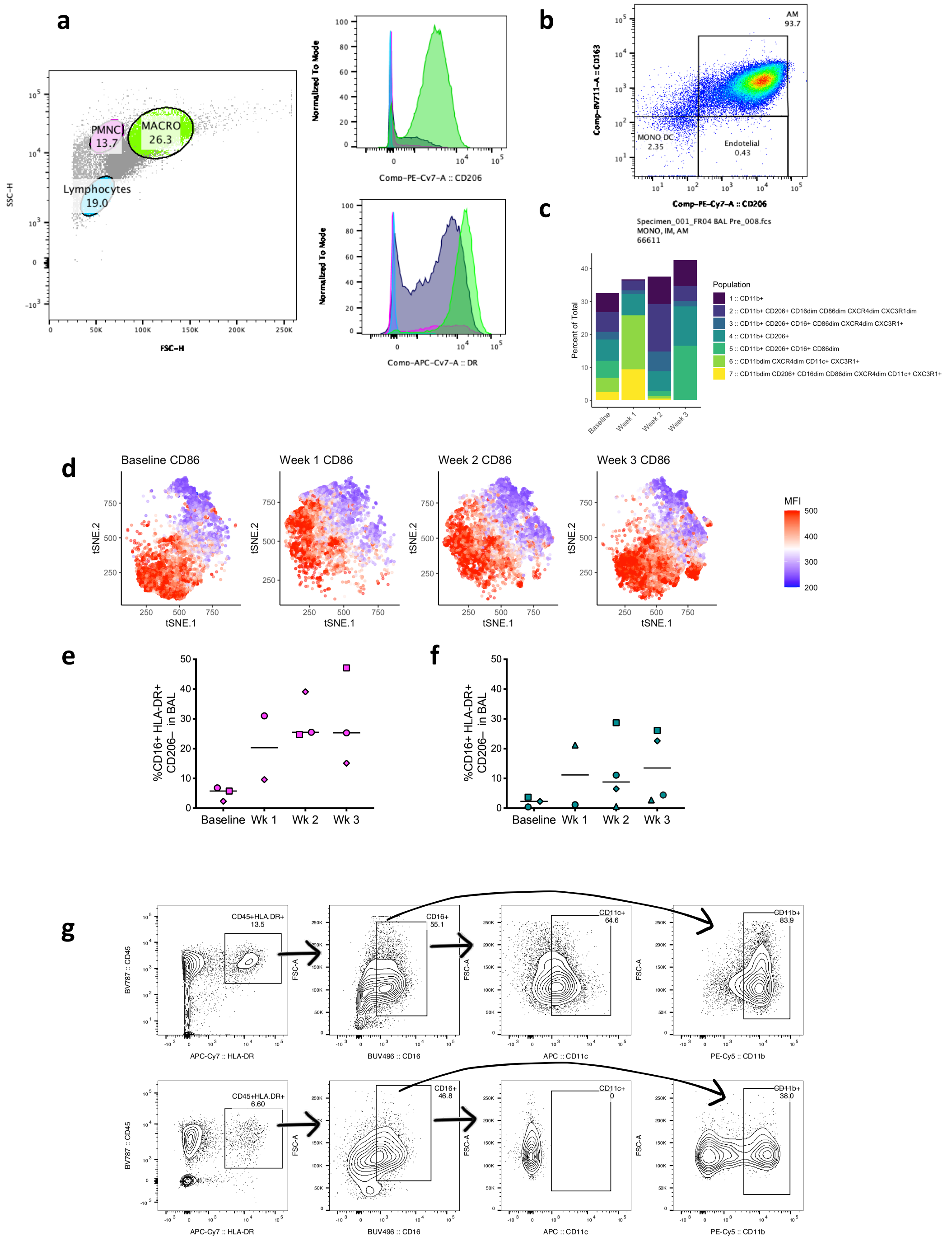

Supplementary Figure 4. Changes in the frequency of alveolar macrophages and infiltrates in BAL during infection by flow cytometric analysis. (**a**) Left panel. Representative light scatter (forward scatter and side scatter) analysis of lymphocytes, macrophages, and polymorphonuclear cells (PMNC) in BAL. Cell populations are located according to their relative size (forward scatter) and granularity (side scatter). Right panels. Overlaid histograms showing fluorescent intensities of CD206 and HLA-DR of lymphocytes, macrophages, and PMNC as shown on the left panels. (**b**) Representative pseudocolor dot plot displaying alveolar macrophages, endothelial cells, monocytes, and/or dendritic cells distinguished by differential expression of CD163 and CD206. (**c**) Range of phenotypes of CD11b+ and CD11c+ myeloid cells in BAL and their change in proportion out of total HLA-DR+ cells over time. (**d**) tSNE plot displaying kinetics of CD86 expression on total HLA-DR+ cells after infection. Percent of CD16+ HLA-DR+ CD206- cells in BAL over time in AGM (**e**) and RM (**f**). (**g**) Representative gating strategy showing classification of CD45+HLA-DR+CD16+ myeloid cells which express either CD11c or CD11b. Top panel showing a sample with CD11c+ cells, and bottom panel showing a sample without CD11c+ cells used to help determine optimal gate position.

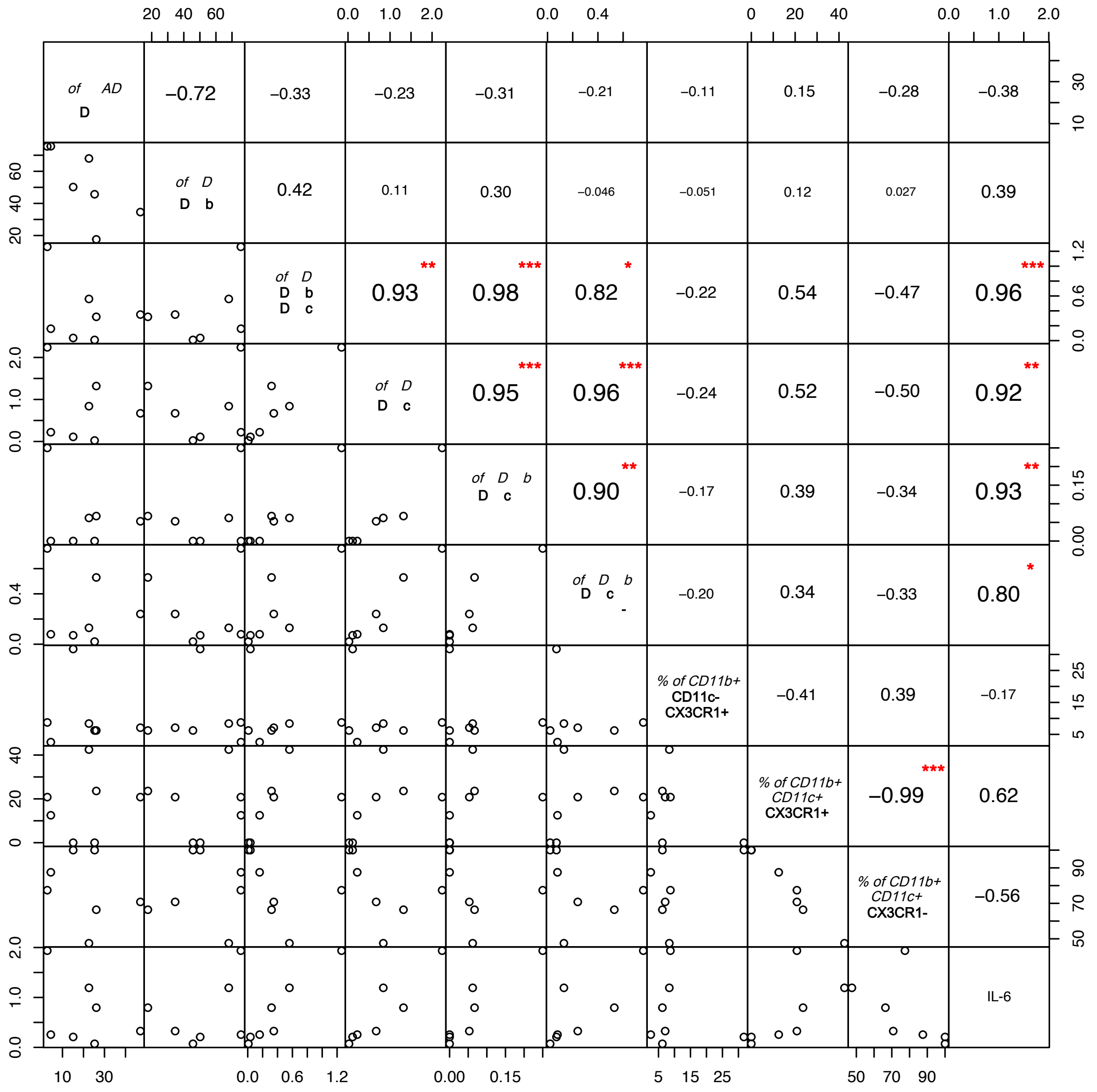

Supplementary Figure 5. **Association of myeloid subsets in the BAL with IL-6 plasma levels**. Correlation plot between myeloid subsets in BAL and levels of IL-6 (pg/mL) in the blood 3 weeks post infection using Pearson's method. Numbers inside boxes on the diagonal right half of the correlation plot represent the coefficient of correlation (r) and asterisks denote significance. One red asterisk corresponds with p <. 05, two red asterisks correspond with p < .01, and three red asterisks correspond with p < .001. Correlation plots in the bottom left display the actual data points.

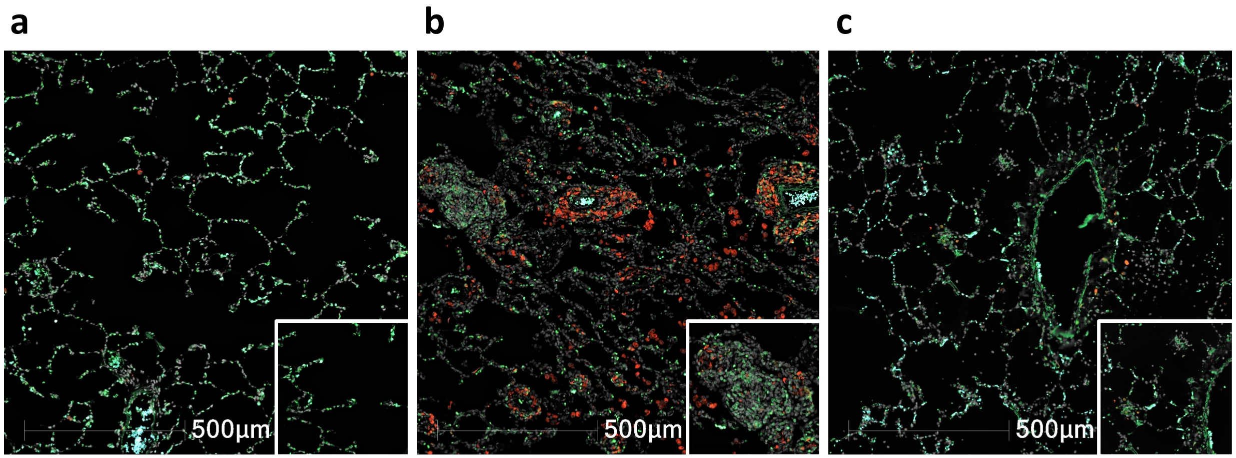

Supplementary Figure 6. Macrophages and CD3 T cells aggregates in the lungs in infected animals. (**a**) FR04: Isolated CD3+ cells scattered throughout the lung. (**b**) GH99: Cell aggregate with mixed population of CD3+ and CD68/CD163+ cells in isolated area of inflammation. (**c**) NC34: Rare, small aggregates of CD3+ cells (White: DAPI, CD68/CD163:red and CD3: blue

a b

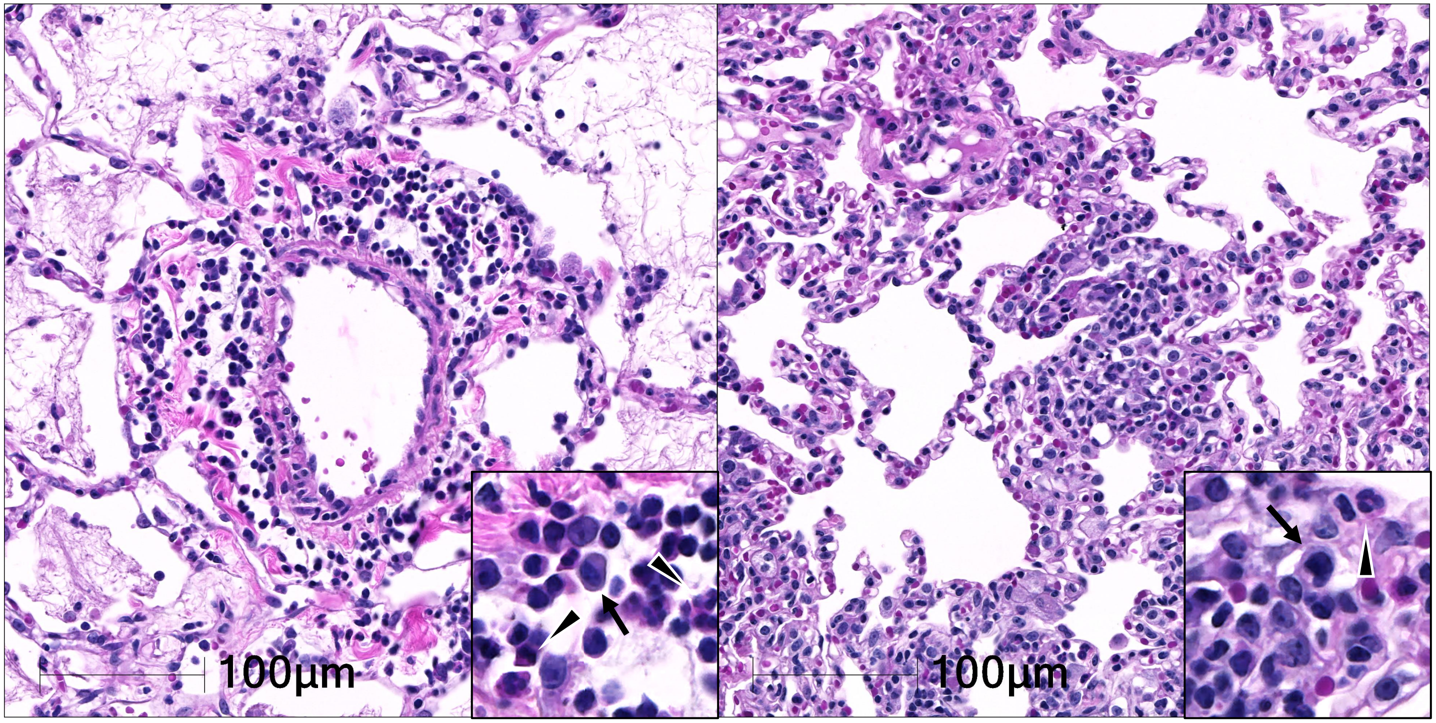

Supplementary Figure 7. **Localization of lymphocytic aggregates in SARS-CoV-2 infection**. Lymphocytes aggregates within the pulmonary interstitium. (**A**) in NC34 (necropsy day 8 post infection; rank = 1, severe) lymphocytic aggregates are perivascular and intermixed with neutrophils and histiocytes. (**B**) In NC38 (necropsy day 28, rank 5, moderate) lymphocytic aggregates are scattered within the alveolar septa and predominately mixed with histiocytes and rare neutrophils. Insets showing the admixture of macrophages (arrows) and neutrophils (arrowheads).

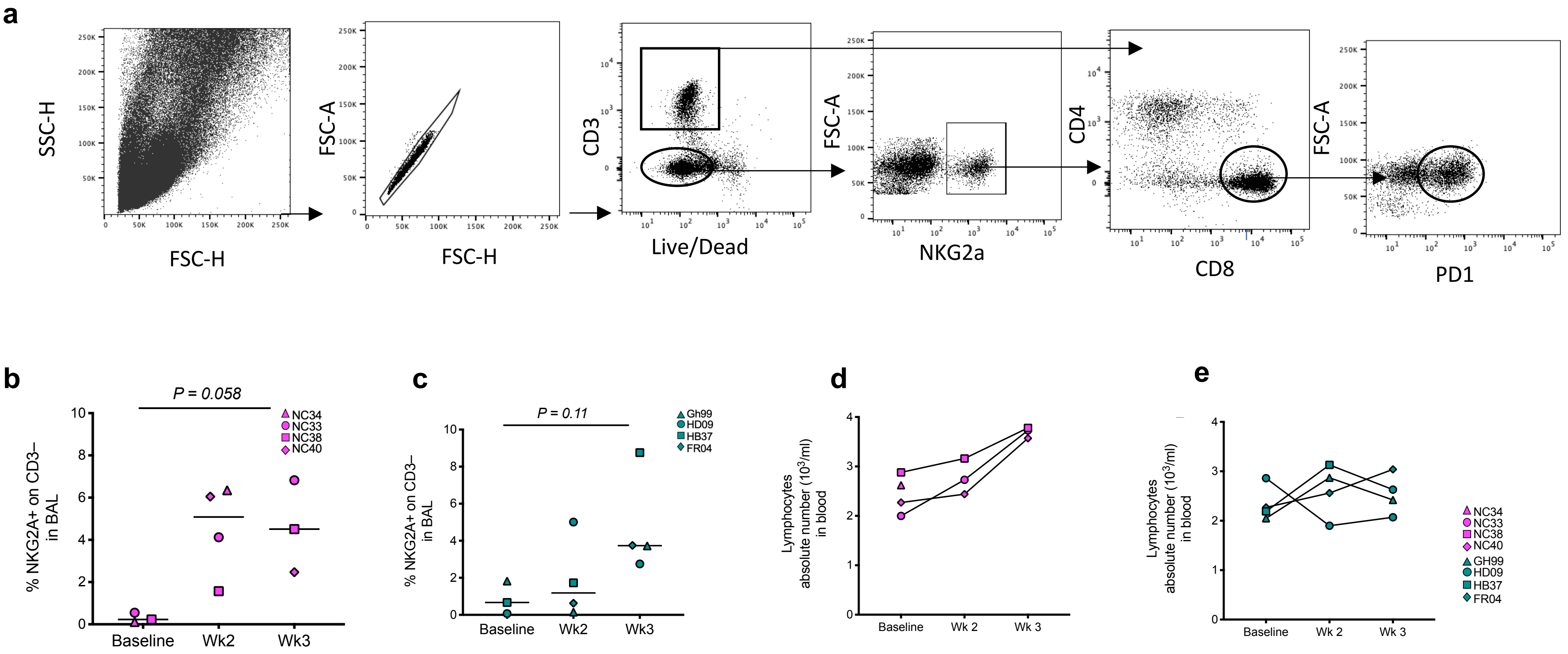

Supplementary Figure 8. NKG2a+ cells and PD-1+ CD8 T cells. (**a**) Gating strategy showing animal FR04 BAL at week 2. BAL was enriched of lymphocytes and NKG2a+ cells and CD8 were gated on the lymphocyte region, singled live CD3 population NKG2a+ cells were then assessed in the CD3 negative gate while CD8 T cells were gated on CD3 positive cells. The frequency of PD-1 was then assessed within the CD8 population. Percent of NKG2a+ on CD3– cells in BAL in AGM (**b**) and RM (**c**). Changes in lymphocyte count in blood before and after infection in AGM (**d**) and RM (**e**).

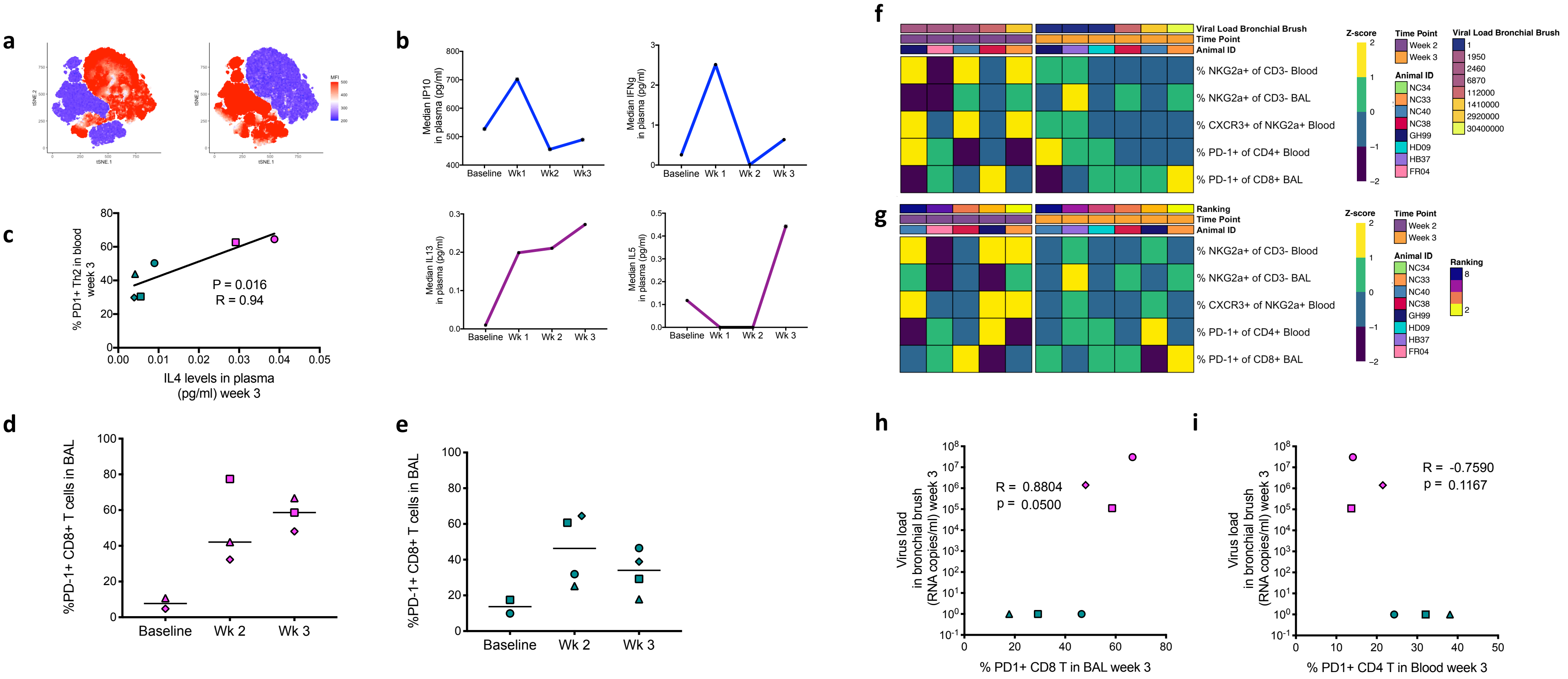

Supplementary Figure 9. SARS-Cov2 associated changes in the quantity and the quality of T cells and NKG2a cells (**a**) CD4 or CD8 T cells were gated from CD3 T cells from all animals over time and data were concatenated. The concatenated data were used to generate tSNE plots depicting regions where CD4 T cells (left panel) and CD8 T cells (right panel) are located. Red coloring indicates the respective location of each cell type. (**b**) Median levels of IP-10, IFN-γ, IL-13, and IL-5 in plasma (pg/mL) from all eight animals over time. (**c**) Correlation between IL-4 levels in the plasma (pg/mL) and percent of PD-1+ Th2 cells in the blood at week 3 post infection. Proportion of PD-1+ CD8 T cells in BAL from AGM (**d**) and RM (**e**) after infection. (**f**) Heatmap showing the scaled frequencies of NKG2a+ and T cell subtypes in relation to viral load in bronchial brushes at weeks two (left panel) and three (right panel) post infection. The Z-score was used to determine distance from the mean and scale coloring. (**g**) Heatmap showing the scaled frequencies of NKG2a+ cells and T cell subtypes in relation to disease ranking at weeks two (left panel) and three (right panel) post infection. The Z-score was used to determine distance from the mean and scale coloring. Correlations between proportion of PD-1+ CD8 T cells (**h**) or PD-1+ CD4 T cells (**I**) and viral load in bronchial brushes three weeks post infection are shown. For c, h and i the Spearman correlation was used.

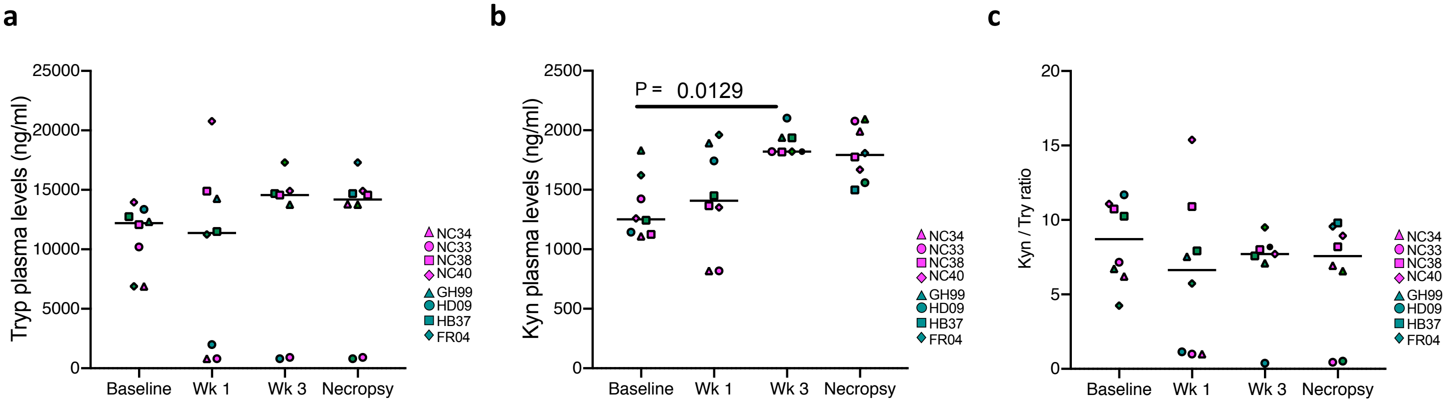

Supplementary Figure 10. SARS-CoV-2 associated changes in IDO activity in plasma over time. (**a**) Levels of tryptophan (Tryp) (**b**) and Kynurenine (Kyn) (ng/ml) in plasma at week 1 and 3 post infection and at necropsy (Kruskal -Wallis test, Dunn's multiple comparison). (**c**) Kyn/ Tryp ratio as a measurement of IDO activity.
