# Supplementary material for: Cellular events of acute, resolving or progressive COVID-19 in SARS-CoV-2 infected non-human primates
Source: Nat Commun. 2020 Nov 27;11:6078. doi: 10.1038/s41467-020-19967-4 (PMC7695721; doi:10.1038/s41467-020-19967-4)
Supplement: Supplementary file 3 — Reporting Summary [file 41467_2020_19967_MOESM3_ESM.pdf]

nature research

Corresponding author(s): Vaccari Monica

Last updated by author(s): 2020 09 14

Reporting Summary

Nature Research wishes to improve the reproducibility of the work that we publish. This form provides structure for consistency and transparency in reporting. For further information on Nature Research policies, see our Editorial Policies and the Editorial Policy Checklist.

Statistics

For all statistical analyses, confirm that the following items are present in the figure legend, table legend, main text, or Methods section.

n/a	Confirmed	
[ ]	[x]	The exact sample size (*n*) for each experimental group/condition, given as a discrete number and unit of measurement
[ ]	[x]	A statement on whether measurements were taken from distinct samples or whether the same sample was measured repeatedly
[ ]	[x]	The statistical test(s) used AND whether they are one- or two-sided<br>*Only common tests should be described solely by name; describe more complex techniques in the Methods section.*
[ ]	[x]	A description of all covariates tested
[ ]	[x]	A description of any assumptions or corrections, such as tests of normality and adjustment for multiple comparisons
[ ]	[x]	A full description of the statistical parameters including central tendency (e.g. means) or other basic estimates (e.g. regression coefficient) AND variation (e.g. standard deviation) or associated estimates of uncertainty (e.g. confidence intervals)
[ ]	[x]	For null hypothesis testing, the test statistic (e.g. *F*, *t*, *r*) with confidence intervals, effect sizes, degrees of freedom and *P* value noted<br>*Give P values as exact values whenever suitable.*
[x]	[ ]	For Bayesian analysis, information on the choice of priors and Markov chain Monte Carlo settings
[x]	[ ]	For hierarchical and complex designs, identification of the appropriate level for tests and full reporting of outcomes
[ ]	[x]	Estimates of effect sizes (e.g. Cohen's *d*, Pearson's *r*), indicating how they were calculated

Our web collection on statistics for biologists contains articles on many of the points above.

Software and code

Policy information about availability of computer code

Data collection	All the softwere to collect data have been indicated in the manuscript in the Material and Methods sections
Data analysis	We have reported code used in material and methods and referred to pubblications in references

For manuscripts utilizing custom algorithms or software that are central to the research but not yet described in published literature, software must be made available to editors and reviewers. We strongly encourage code deposition in a community repository (e.g. GitHub). See the Nature Research guidelines for submitting code & software for further information.

Data

Policy information about availability of data

All manuscripts must include a data availability statement. This statement should provide the following information, where applicable:

- Accession codes, unique identifiers, or web links for publicly available datasets
- A list of figures that have associated raw data
- A description of any restrictions on data availability

All the data will be made available upon pubblication

Field-specific reporting

Life sciences study design

All studies must disclose on these points even when the disclosure is negative.

Sample size	This aim of the study was to describe changes in immune responses upon SARS-COv2 infection\ in a first ever conducted study in NHP. 8 animals were allocated in total 4 for each species, or variable group. This number is sufficient to determine changes overtime - (before and after infection) and 8 animals comparison was used when no difference was found between groups. We have provided all the statistic for all groups (Supplementary Tables).
Data exclusions	n/a No data were excluded
Replication	We have made available all the informations needed . Briefly all our samples data and analyses were performed following stick SOP applayes to many studies and run by experience core personal.
Randomization	All groups of challenges were randomized based on gender and species. All animals were then exposed to the same virus stock.
Blinding	As the first experiment in C Blinding was irrelevant as all animals were infected and treated the same way<br>As the first experiment in SARS-COV2, all animals received the same virus and comparison were done before and after the challenge.

Reporting for specific materials, systems and methods

We require information from authors about some types of materials, experimental systems and methods used in many studies. Here, indicate whether each material, system or method listed is relevant to your study. If you are not sure if a list item applies to your research, read the appropriate section before selecting a response.

Materials & experimental systems

n/a	Involved in the study	
☐	☒	Antibodies
☒	☐	Eukaryotic cell lines
☒	☐	Palaeontology and archaeology
☐	☒	Animals and other organisms
☒	☐	Human research participants
☒	☐	Clinical data
☒	☐	Dual use research of concern

Methods

n/a	Involved in the study	
☒	☐	ChIP-seq
☐	☒	Flow cytometry
☒	☐	MRI-based neuroimaging

Antibodies

Antibodies used	We used commercial antibodies that are described in Supplementary Table 10 and in the Mesoscale and Immunoistochemestry section.
Validation	All antibodies used were commercial and tested in NHP. https://www.nhpreagents.org/ was used to look up for antibodies reacting to our species. Both Beckton Dikinson and Biolegend reports reactivity to Rhesus on their websites and on the specific antibodies as Reactive to humans rhesus etc.

Animals and other organisms

Policy information about studies involving animals; ARRIVE guidelines recommended for reporting animal research

Laboratory animals	We have used Indian Rhesus macaques and African green monkeys, as described in Supplementary Table 1 animals wer all oldwr tha 13 and both females and male
Wild animals	AGM were cought 1 year prior the study and were negative for SIV. The animals were kept ion corollaries for at least a year untill the study and euthanised at the end of the study.
Field-collected samples	n/a No Field collected samples were used in this study
Ethics oversight	IACUC approved the protocol as stated in material and methods first paragraph The Institutional Animal Care and Use Committee of Tulane University reviewed and approved all the procedures for this study

Note that full information on the approval of the study protocol must also be provided in the manuscript.

Flow Cytometry

Plots

Confirm that:

- [x] The axis labels state the marker and fluorochrome used (e.g. CD4-FITC).
- [x] The axis scales are clearly visible. Include numbers along axes only for bottom left plot of group (a 'group' is an analysis of identical markers).
- [x] All plots are contour plots with outliers or pseudocolor plots.
- [x] A numerical value for number of cells or percentage (with statistics) is provided.

Methodology

Sample preparation	Sample preparation is described Freshly obtained PBMCs and cells obtained from BAL were stained with appropriate antibody cocktails and incubated for 30 minutes at room temperature.
Instrument	Instrument is described We used a FACS Aria Fusion, BD instrument
Software	Flow jo was used as described
Cell population abundance	We did not sort cells
Gating strategy	Gating strategy are provided in Supplementary Figure 2, 4m, and 8 All gatings were based on live cells within the FSH SSCH gates and the positive populations were based on FMO previusly obained on not stained PBMCs and on the negative and positive beads

- [x] Tick this box to confirm that a figure exemplifying the gating strategy is provided in the Supplementary Information.
